# Neurons in the Dorsomedial Hypothalamus Promote, Prolong, and Deepen Torpor in the Mouse

**DOI:** 10.1523/JNEUROSCI.2102-21.2022

**Published:** 2022-05-25

**Authors:** Michael Ambler, Timna Hitrec, Andrew Wilson, Matteo Cerri, Anthony Pickering

**Affiliations:** ^1^Anaesthesia, Pain, & Critical Care Sciences, School of Physiology, Pharmacology, & Neuroscience, University of Bristol, Bristol, BS8 1TD, United Kingdom; ^2^Department of Biomedical & Neuromotor Sciences, University of Bologna, Bologna, 40127, Italy

**Keywords:** dorsomedial, hypothalamus, metabolism, thermoregulation, torpor, TRAP

## Abstract

Torpor is a naturally occurring, hypometabolic, hypothermic state engaged by a wide range of animals in response to imbalance between the supply and demand for nutrients. Recent work has identified some of the key neuronal populations involved in daily torpor induction in mice, in particular, projections from the preoptic area of the hypothalamus to the dorsomedial hypothalamus (DMH). The DMH plays a role in thermoregulation, control of energy expenditure, and circadian rhythms, making it well positioned to contribute to the expression of torpor. We used activity-dependent genetic TRAPing techniques to target DMH neurons that were active during natural torpor bouts in female mice. Chemogenetic reactivation of torpor-TRAPed DMH neurons in calorie-restricted mice promoted torpor, resulting in longer and deeper torpor bouts. Chemogenetic inhibition of torpor-TRAPed DMH neurons did not block torpor entry, suggesting a modulatory role for the DMH in the control of torpor. This work adds to the evidence that the preoptic area of the hypothalamus and the DMH form part of a circuit within the mouse hypothalamus that controls entry into daily torpor.

**SIGNIFICANCE STATEMENT** Daily heterotherms, such as mice, use torpor to cope with environments in which the supply of metabolic fuel is not sufficient for the maintenance of normothermia. Daily torpor involves reductions in body temperature, as well as active suppression of heart rate and metabolism. How the CNS controls this profound deviation from normal homeostasis is not known, but a projection from the preoptic area to the dorsomedial hypothalamus has recently been implicated. We demonstrate that the dorsomedial hypothalamus contains neurons that are active during torpor. Activity in these neurons promotes torpor entry and maintenance, but their activation alone does not appear to be sufficient for torpor entry.

## Introduction

Torpor is the naturally occurring hypothermic, hypometabolic, and hypoactive component of hibernation, which can be prolonged (in seasonal hibernators) or brief (in daily heterotherms, such as the mouse). It serves as an adaptive response to relative energy deficit: a controlled reduction in metabolic demand in response to reduced availability of substrate. During torpor, body temperature typically runs a few degrees above ambient temperature, which in hibernating arctic ground squirrels results in core temperatures as low as −2.9°C (Barnes, 1989). Metabolic rate falls to between 1% and 5% of euthermic rates with similar reductions in heart and respiratory rates (Heldmaier et al., 2004).

The mechanisms controlling this profound deviation from normal physiology are not known, but recent work has indicated a role for the preoptic area of the hypothalamus (POA) in generating a hypothermic, hypometabolic, bradycardic state similar to daily torpor in the mouse (Hrvatin et al., 2020; Takahashi et al., 2020; Zhang et al., 2020). These neurons are active during natural fasting-induced daily torpor (Hrvatin et al., 2020; Zhang et al., 2020); are necessary for the full expression of daily torpor (Hrvatin et al., 2020; Takahashi et al., 2020; Zhang et al., 2020); and express *Adcyap1* (Hrvatin et al., 2020) and/or pyroglutamylated RFamide peptide (Takahashi et al., 2020), and/or estrogen receptors (Zhang et al., 2020), with evidence of overlap in these markers. These studies suggest that activation of a projection from the POA to the dorsomedial hypothalamus (DMH) contributes to the generation of this torpor-like state, although projections from the POA elsewhere, for example to arcuate, may also contribute (Hrvatin et al., 2020; Zhang et al., 2020).

Projections from the POA to DMH are also involved in thermoregulation outside of the context of torpor. Warm-activated neurons in the ventral part of the lateral POA project to the DMH and inhibit thermogenesis and locomotion (Zhao et al., 2017). Similarly, warm-activated neurons in the ventromedial POA that express *Adcyap1* and BDNF project to the DMH and suppress thermogenesis (Tan et al., 2016). These thermogenesis inhibiting warm-activated POA to DMH projections that play a role in thermoregulation are GABAergic (Tan et al., 2016; Zhao et al., 2017), in contrast to the predominantly glutamatergic projections that are implicated in torpor (Hrvatin et al., 2020; Takahashi et al., 2020). In addition to an established role in thermoregulation, the DMH also regulates energy balance and food intake (Yang et al., 2009; Bi et al., 2012; Jeong et al., 2015), heart rate (Piñol et al., 2018), and plays a role in adjusting circadian rhythms in response to food availability (Chou et al., 2003; Gooley et al., 2006; Mieda et al., 2006). Hence, the DMH is well positioned to play a role in the regulation of torpor, and there is evidence to support its activation during daily torpor in the mouse (Hitrec et al., 2019).

We hypothesized that the DMH plays a role in the control of torpor. To test this hypothesis, we used activity-dependent targeting of neurons (DeNardo et al., 2019) within the DMH that were active during natural torpor bouts in female mice. Using this approach, we first labeled neurons within the hypothalamus that were active during torpor. Next, we expressed excitatory or inhibitory DREADDs (Roth, 2016) in DMH neurons that were active during torpor, and evaluated the effects of activating these receptors on the propensity to enter torpor during calorie restriction.

## Materials and Methods

### Mice

All studies had the approval of the local University of Bristol Animal Welfare and Ethical Review Board and were conducted in accordance with the UK Animals (Scientific Procedures) Act. Female *TRAP2* (Allen et al., 2017; DeNardo et al., 2019), *Ai14* (Madisen et al., 2010), and C57BL/6J mice, at least 8 weeks of age with mean body weight 22.9 ± 2.8 g on experiment entry were used. Female mice were chosen because of their increased propensity to undergo torpor (Swoap and Gutilla, 2009). Mice were maintained on a reversed 12 h light/dark cycle with lights off at 08:30; hence, lights off was assigned as zeitgeber time (ZT) 0. Ambient temperature was 20.9 ± 0.4°C. Mice had free access to water and standard mouse chow (LabDiet), except during periods of calorie restriction when food was limited as detailed below. They were housed in groups of up to four.

A homozygous breeding colony was established from heterozygous *TRAP2* mice kindly donated by the Liqun Luo laboratory in Stanford University, California. The strain is now available via The Jackson Laboratory (www.jax.org/strain/030323). The *TRAP2* mouse line carries the gene for Cre-ERT2 under the fos promoter leading to Cre-ERT2 expression in active neurons. Homozygous *Ai14* mice were obtained from the University of Bristol in-house colony, having been originally purchased from The Jackson Laboratory (www.jax.org/strain/007908). The *Ai14* mouse carries a floxed gene encoding a red fluorophore (tdTomato) knocked into the *Gt(ROSA)26Sor* locus. This requires the action of Cre to remove a stop codon to allow tdTomato expression. Breeding pairs were established with homozygous *TRAP2* and homozygous Ai14 mice to generate *TRAP:Ai14* double-heterozygous offspring. The resultant double-transgenic *TRAP:Ai14* mice produce red fluorescent tdTomato protein in neurons that were active during the time period defined by the injection of 4-hydroxytamoxifen (4-OHT).

### Viral vectors

Three viral vectors were used:

AAV2-hSyn-DIO-hM3Dq-mCherry was a gift from Bryan Roth (Addgene viral prep #44361-AAV2; www.addgene.org/44361) (Krashes et al., 2011); 4.6 × 10^12^ viral genome copies per ml. This vector delivered a Cre-dependent mCherry-tagged excitatory DREADD gene under the human synapsin promoter. It was mixed in a 4:1 ratio with the EGFP-expressing vector (used to identify the injection site), giving a final titer for this vector of 3.7 × 10^12^ viral genomes per ml.

AAV2-hSyn-DIO-hM4Di-mCherry was a gift from Bryan Roth (Addgene viral prep #44362-AAV2; www.addgene.org/44362) (Krashes et al., 2011); 2 × 10^13^ viral genome copies per ml. This vector delivered a Cre-dependent mCherry-tagged inhibitory DREADD gene under the human synapsin promoter. It was mixed in a 1:2 ratio with the EGFP-expressing vector, and the resulting vector mixture further diluted in a 10-fold with sterile PBS, giving a final titer of 6.7 × 10^11^ viral genomes per ml.

pAAV2-CMV-PI-EGFP-WPRE-bGH was a gift from James M. Wilson (Addgene viral prep #105530-AAV2; www.addgene.org/105530); 7 × 10^12^ viral genome copies per ml. This vector delivered the gene coding for EGFP under the ubiquitous CMV promoter. This vector was used to confirm the localization of injection because the expression of mCherry fluorescence in the two vectors above is contingent on successful TRAPing (and so would not be visible if the injected area is not TRAPed).

### Stereotaxic injection of viral vectors

Mice were anesthetized with ketamine (70 mg/kg i.p.) and medetomidine (0.5 mg/kg i.p.). Depth of anesthesia was assessed and monitored by loss of hind paw withdrawal reflex. Additional intraperitoneal injections of anesthetic were administered as needed to maintain surgical depth of anesthesia. Core temperature was maintained using a servo-controlled heat pad and a rectal temperature probe (Harvard Apparatus). The planned incision site was shaved, and skin cleaned with iodine solution and sterile surgical technique was used throughout. Anesthetized mice were placed in a stereotaxic frame, the head was fixed in atraumatic ear bars, and skull position maintained horizontal by an incisor bar (David Kopf Instruments).

Microcapillary pipettes were made from microcapillary glass (Sigma) on a vertical pipette puller (Harvard Apparatus). Pipettes were filled with mineral oil; then vector was back-filled using a robotic microinjector (Nano-W wireless capillary microinjector, Neurostar), producing a visible vector–mineral oil interface. The scalp was incised in the midline and burr holes made bilaterally at bregma −1.8 mm, lateral ±1 mm with a drill attachment (Neurostar). The microcapillary pipette was inserted at an angle of 8° toward the midline. Bilateral injections were made at depths of 5 and 4.75 mm relative to the surface of the brain. Each injection was 180 nl and was delivered at a rate of 100 nl/minute. The injection pipette remained in place for 1 min after the first injection and for 5 min after the second before removing.

Following vector injections, the wound was closed with nonabsorbable suture and dressed with antibacterial wound powder. Anesthesia was reversed with atipamezole (1 mg/kg i.p, Antisedan, Zoetis). Buprenorphine was administered for analgesia (0.1 mg/kg s.c., Vetergesic, Ceva Animal Health). Mice were recovered on a heat pad, then housed individually for 3 d following surgery and monitored daily until they recovered to baseline weight.

### Torpor induction

Before torpor induction, mice were moved from the home cage to a custom-built 32 × 42 × 56 cm cage, divided into four quadrants (16 × 21 × 56 cm), into which each mouse was individually placed. This cage was designed to allow up to 4 animals to be monitored simultaneously using a single thermal imaging camera placed directly above. The Perspex separating each quadrant was clear and had ventilation holes at 2 cm from the floor height to allow interaction between mice in neighboring quadrants, while preventing huddling.

**Figure 1.**
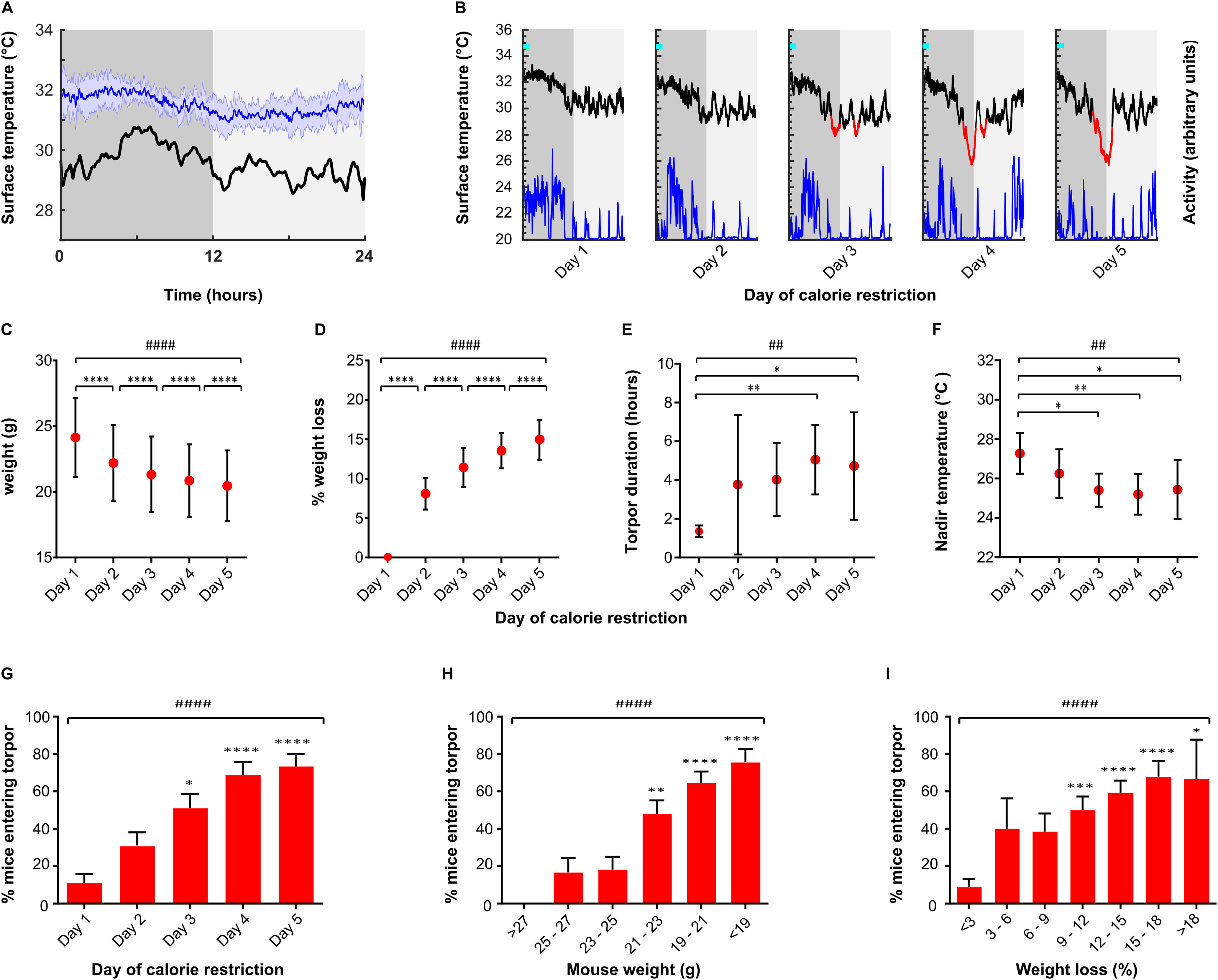
Characteristics of torpor induced by calorie restriction. ***A***, Mean mouse surface temperature across the 24 h day cycle housed at 21°C (*n* = 7 female mice recorded for 3 consecutive days, blue line). Shaded area represents SD for that ZT. Torpor threshold (black line) represents 4 SDs from the mean temperature at that ZT. ***B***, Example profile from single mouse undergoing torpor by 5 d calorie restriction. Mice receive a single meal at lights off (ZT0, cyan marker), providing ∼70% of the ad libitum daily intake. Surface temperature (black/red line) is measured using infrared thermography, activity (blue) derived from thermal imaging video. Torpor (red line) defined as a period during which surface temperature remained 4 SDs below the mean for that time of day for a period of at least 1 h. ***C***, ***D***, Weight loss by day of calorie restriction. ***E***, ***F***, Torpor bout duration and nadir surface temperature by day of calorie restriction. ***G***, Proportion of mice entering torpor for each consecutive day of calorie restriction. ***H***, Proportion of mice entering torpor by absolute weight. ***I***, Proportion of mice entering torpor by % weight loss from first day of calorie restriction. Tests used were Kruskal-Wallis for continuous variables and Friedman test for binary variables. Main effect for day of calorie restriction or weight loss: ^#^*p* < 0.05; ^##^*p* < 0.01; ^###^*p* < 0.001; ^####^*p* < 0.0001. Individual comparisons: *p < 0.05; **p < 0.01; ***p < 0.001; ****p < 0.0001, respectively. ***G-I***, Individual comparisons are relative to mice on day 1. *n* = 45 trials in 30 female mice, 5 consecutive days calorie restriction at 21°C ambient temperature with no chemogenetic manipulation.

A calorie restriction protocol was used to increase the likelihood of torpor induction (see Fig. 1) (van der Vinne et al., 2018). Mice were weighed and given a single daily meal placed directly onto the floor of the cage at lights off (ZT0) for 5 consecutive days. The meal consisted of one pellet (2.2 g) of feed (EUROdent Diet 22%, irradiated, 5LF5). This provides 8 kcal per day, which is ∼70% of the estimated unrestricted daily intake for a mouse of this size (Subcommittee on Laboratory Animal Nutrition and Board on Agriculture, 1995).

Mouse surface temperature was recorded using an infrared thermal imaging camera placed above the cage (Flir C2, www.flir.co.uk). Baseline recordings of mouse surface temperature were taken for a period of 3 d at an ambient temperature of 21°C. During this time, mice had free access to food and were therefore not expected to enter torpor. These measurements were then used to generate a daily profile mean and an SD of the temperature fluctuations across the diurnal cycle (in 1 min bins).

### TRAP:Ai14 tagging

Female *TRAP:Ai14* mice were entered into the calorie restriction protocol and were habituated to daily vehicle injection (chen oil, see below) at ZT7, before receiving 4-OHT on day 3, again at ZT7. Approximately half the animals entered torpor within 3 h of 4-OHT (50 mg/kg), and those that did not were used as controls.

### Chemogenetic targeting of DMH neurons

Adeno-associated viral vectors were used to deliver either Cre-dependent excitatory (hM3Dq) or inhibitory (hM4Di) DREADD transgenes into the DMH of TRAP2 mice as described above. After at least 2 weeks recovery, mice were entered into the calorie restriction torpor induction protocol during which they were habituated to daily vehicle injection (chen oil, see below) at ZT7 before receiving 4-OHT on day 5, again at ZT7.

Following a further 2 weeks to allow return to baseline weight and to allow expression of the DREADD protein, mice entered a randomized, crossover design, calorie restriction trial. During this trial, each mouse was randomly assigned to receive either daily clozapine-N-oxide (CNO) (5 mg/kg, i.p) or daily saline injections (0.9%, 5 ml/kg, i.p) at ZT7, on each of the 5 d of calorie restriction. The occurrence and depth of torpor were monitored with surface thermography. Following this first arm of the study, and after at least 5 d with free access to food, the process was repeated with mice that initially received CNO now receiving saline, and vice versa.

To control for a potential influence of CNO on torpor, WT mice (without a vector injection) underwent the same CNO versus saline crossover trial protocol. To control for an influence of basal DMH activity on torpor, further *TRAP2* mice underwent DMH vector injection followed by 4-OHT injection at ZT7 in the home cage with free access to food. These mice were then entered into the calorie restriction protocol with CNO/saline dosing. Torpor duration and nadir temperature reached were recorded daily for each animal.

Mouse surface temperature was identified by extracting the maximum temperature value from ROIs within each frame that correspond to each individual mouse's compartment within the cage. Peak temperature data were extracted from the infrared video using Flir ResearchIR software version 4.40.9 (www.flir.co.uk), and further filtered and analyzed using MATLAB 2019a (www.mathworks.com). The data processing stream was as follows: to limit noise and movement artifact, data points <20°C or >40°C were removed; data were then interpolated and resampled at 1 Hz, using the MATLAB *interpl* function; finally, a moving average filter function was applied with a 360 data point window, using the MATLAB *smooth* function. Mouse activity data were derived from the thermal imaging video, extracted using Ethovision XT software (www.noldus.com).

### Histology

#### TRAP:Ai14 tagging

Mice were culled by terminal anesthesia (pentobarbitone, Euthatal, 175 mg/kg,) a minimum of 4 weeks from the time of 4-OHT injection, to allow expression of tdTomato. They were transcardially perfused with 10 ml heparinized 0.9% NaCl (50 units/ml) followed by 20 ml of 10% neutral buffered formalin. Brains were removed and stored in fixative solution for 24 h at 4°C before being transferred to 20% sucrose in 0.1 m PB, pH 7.4, and again stored at 4°C. Brains were sectioned at 40 μm thickness into a 1:3 series on a freezing microtome, transferred to 0.1 m PB containing 1:1000 sodium azide. Sections were imaged using a Leica DMI6000 widefield microscope and 0.75 numerical aperture, 20× magnification objective, excitation filter 546/10 nm, dichroic mirror 560 nm, emission filter 580/40 nm.

Masks for ROIs were taken from the Mouse Brain Atlas (Franklin and Paxinos, 2007). These masks were then digitally applied to each of the widefield images so that the same size and shape area of interest were used for cell counting across animals. The DMH, posterior hypothalamus (PH), and arcuate nucleus (Arc) were defined by their atlas boundaries. The preoptic area mask was defined dorsally by the anterior commissure, ventrally by the ventral surface of the brain, and laterally by the lateral extent of the ventrolateral preoptic nucleus. Hence, the preoptic area as defined here included the medial and lateral parts of the medial preoptic nucleus, the ventromedial and ventrolateral preoptic nuclei, the medial and lateral preoptic areas, and parts of the strio-hypothalamic, the septo-hypothalamic, the median preoptic, and the periventricular nuclei (Franklin and Paxinos, 2007). An automated image processing pipeline was used to count c-fos-positive nuclei. This involved background subtraction with a rolling ball radius of 50 pixels. Labeled cells were identified by applying a threshold to the image that identified regions with brightness 3 SDs greater than the mean background. Overlapping regions were separated using the watershed method. Highlighted areas were then filtered by size (50-2000 μm^2^) and counted in ImageJ.

#### Multiplex RNA ISH

Mice were culled by terminal anesthesia with intraperitoneal pentobarbitone (175 mg/kg, Euthatal). Fresh frozen tissue was prepared, and 15 µm coronal sections were cut using a cryostat.

Every third section was taken for standard immunohistochemistry to confirm appropriate injection targeting and expression of the DREADD-mCherry fusion protein using a rabbit anti-mCherry primary (Biovision 5993, 1:2000), and donkey anti-rabbit secondaries (AlexaFluor-594, 1:1000). Sections were imaged using a Zeiss Axioskop II inverted microscope with a CooLED pE-100 excitation system, excitation filter 546/12 nm, dichroic mirror 580 nm, emission filter 590 nm.

For RNAscope (Acdbio), mounted sections were fixed in fresh 10% neutral buffered formalin solution for 1 h at room temperature, then dehydrated using a sequence of increasing concentrations of ethanol (50%-100%). One slide containing the DMH at bregma −1.94 mm was selected from each animal to be processed for multiplex RNAscope for a total of 12 probes (Table 1). Dehydrated sections were air-dried for 5 min at room temperature. Slides were then treated with a protease (Protease IV) for 30 min at room temperature. The 12 RNA probes were then applied to each slide and hybridized for 2 h at 40°C in a humidified chamber. After hybridization, slides were washed in wash buffer twice for 2 min at room temperature. Amp 1 was applied to each slide and incubated in a humidified oven at 40°C for 30 min, then again washed twice. The process was repeated for two further amplification steps using Amp 2, then Amp 3.

**Table 1.** RNA Target probes used across the 12 rounds of ISH*^a^*

Fluorophore label	Round 1	Round 2	Round 3	Round 4
AF488	Leptin receptor B (catalog #402731)	NPY (catalog #313321)	ChAT (catalog #408731)	mCherry (catalog #431201)
Atto550	Kappa opioid receptor (catalog #316111)	TRH (catalog #4368110	Galanin (catalog #400961)	VGLUT2 (catalog #319171)
Atto647	NPY receptor 1 (catalog #4270210)	Orexin (catalog #490461)	VGAT (catalog #319191)	NeuN (catalog #313311)

*^a^*Three RNA targets were imaged in each round, alongside DAPI. The fluorophores were cleaved and then attached to the next round of targets across four rounds. NPY, Neuropeptide Y; TRH, thyrotropin releasing hormone.

Next, HiPlex Fluorophore (targeting RNA probes 1-3) was added and incubated in a humidified oven at 40°C for 15 min, then again washed twice for 2 min each time at room temperature. Finally, DAPI was applied to the sections for 30 s at room temperature, followed by Prolong Gold antifade mountant (https://www.thermofisher.com/), and a coverslip was applied. Sections were then imaged using a confocal microscope (see below).

After imaging the DAPI, AF488, Atto550, and Atto647 channels, coverslips were removed by soaking in 4× saline sodium citrate (https://www.thermofisher.com/) for 30 min. Cleaving solution (https://acdbio.com/) was applied to each section and incubated at room temperature for 15 min to remove the fluorophores from the RNA probe amplifiers, followed by two washes in PBST (PBS with 0.5% Tween). This cleaving process was repeated once.

The entire protocol, from the point of applying the fluorophores to imaging and cleaving, was then repeated 3 further times, with fluorophores targeting probes 4-6, 7-9, then 10-12 on each successive round. On each occasion, DAPI plus three RNA target probes were imaged until all 12 targets had been processed.

Sections for RNAscope were imaged using a Leica SP8 AOBS confocal laser scanning microscope attached to a Leica DMi8 inverted epifluorescence microscope using HyD detectors (“hybrid” SMD GaAsP detectors) with 405 nm diode and white light lasers. A 40× oil immersion lens with NA of 1.3 (Leica HC PLAPO CS2 lens) was used. Laser settings are shown in Table 1.

The images generated from each round were *z* projection compressed in ImageJ (Schindelin et al., 2012); then proprietary software (www.acdbio.com) was used to align the images from each round for each animal, based on the DAPI staining. Aligned images were then processed in ImageJ first applying a 50 pixel radius rolling ball background subtraction. Then, in order to minimize the effects of autofluorescence, for each fluorescence channel the images generated across the 4 rounds (each labeling a different RNA target probe) were stacked and a median projection was generated. This background fluorescence signal was then subtracted from each image prior to analysis.

Each DAPI-stained nucleus was identified and marked with a 12-µm-diameter area of interest. This created a map of all the nuclei within the imaged section, which could then be projected onto the fluorescence images from each round of RNA target visualization for that section. DAPI-stained nuclei lining the third ventricle were excluded as these were assumed to represent ependymal cells. Where two DAPI nuclei appeared to overlap, both were excluded on the basis that it would not be possible to distinguish to which nucleus a given RNA probe signal was attached. Nuclei expressing each RNA target were identified manually. From each section, the region immediately lateral to the dorsal part of the third ventricle at bregma −1.94 mm was imaged. This included the dorsal and compact parts of the DMH. The area scanned measured 387 × 387 × 10 µm (see Fig. 8).

### Drug preparation

#### Vehicle

The vehicle for 4-OHT was chen oil, composed of four parts sunflower seed oil and one part castor oil.

#### 4-OHT

The z-isomer of 4-OHT is the active isomer (www.tocris.com). It was dissolved in chen oil using the following method (Guenthner et al., 2013). First, 4-OHT was dissolved in neat ethanol at 20 mg/ml by shaking at 400 rpm and 37°C for 30-60 min until fully dissolved. Two parts chen oil for every one part ethanol was then added, and the ethanol was evaporated off using a vacuum centrifuge leaving a final solution of 10 mg/ml in chen oil. Drug was prepared on the day of use, and if not used immediately, was kept in solution in the oil by shaking at 400 rpm at 37°C. Once in solution, the drug was protected from light.

#### CNO

CNO was dissolved at 1 mg/ml in sterile water at room temperature. Aliquots were stored protected from light for up to 1 week at room temperature.

### Experimental design and statistical analyses

During baseline recordings in fed mice (*n* = 7 female mice recorded for 3 consecutive days), we found that surface temperature frequently dropped below 3 SDs from the mean, but infrequently dropped below 4 SDs from the mean (see Fig. 1*A*,*B*). Adding a time criterion of 60 min duration for the period spent outside of the normal thermoregulatory range optimized the specificity for torpor detection and helped distinguish torpor from sleep: no ad libitum fed mice remained 4 SDs below the mean for this length of time, yet visually obvious torpor bouts in calorie-restricted mice were reliably detected. Hence, we defined torpor as any period during which mouse surface temperature remained 4 SDs below the mean for that time of day for a period of at least 1 h.

Data were analyzed using GraphPad Prism (version 6.07). To assay chemogenetic promotion or inhibition of torpor, data from calorie restriction periods in which mice received CNO were compared with data from calorie restriction periods in which the same mice received saline. Data for daily time spent in torpor and nadir temperature were analyzed using two-way repeated-measures ANOVA with Holm–Sidak's multiple comparisons test. Analysis of the probability of torpor during calorie restriction used Friedman or Kruskal–Wallis test with Dunn's multiple comparisons. Comparison of the total number of days with torpor, day of first torpor entry, and weight at first torpor entry when calorie-restricted mice received CNO versus saline were analyzed using paired-samples *t* test or Wilcoxon matched pairs signed rank test. Normality was assessed by Kolmogorov–Smirnov test. Data are presented as mean ± SD for normally distributed data, or else median [interquartile range]. Where the day of torpor emergence was analyzed, if no bout appeared by the end of 5 d calorie restriction, for analyses torpor was assumed to occur on day 6.

## Results

### Torpor induction by calorie restriction

Before chemogenetic manipulation of torpor-TRAPed neurons, the calorie restriction protocol generated torpor bouts on at least 1 day in 97% of 45 trials in 30 mice. Mean nadir surface temperature during torpor was 25.3 ± 1.3°C compared with a mean nadir of 30.0 ± 0.7°C in mice held at the same ambient temperature (21°C) with free access to food (*t*_(11)_ = 9.40, *p* < 0.0001). Entry into torpor occurred in the second half of the lights off period, with the median time of entry into torpor occurring at ZT 9.76 [8.18-10.83] hours after lights off.

Torpor duration increased from median 1.27 h [1.09-1.65] on day 1 to 4.47 h [1.90-6.77] on day 5 (Kruskal–Wallis test, *H*(5) = 18.05, *p* < 0.01) (Fig. 1*E*). The increase in torpor duration was associated with torpor occurring increasingly early in the day, from a median 12.42 h [11.21-15.13] from lights off on day 1 to median 9.11 h [7.87-10.77] on day 5 (Kruskal-Wallis *H*(5) = 20.20, *p* < 0.001). The nadir temperature reached during torpor decreased with increasing days of calorie restriction from 27.3 ± 1.0°C on day 1 to 25. 4 ± 1.5°C on day 5 (one-way ANOVA *F*_(4,102)_ = 4.717, *p* < 0.01) (Fig. 1*F*). Activity of the mice, derived from the thermal imaging video, reduced to a minimum during torpor, and increased during arousal.

Mice lost weight across the 5 d of calorie restriction (Fig. 1*C*,*D*). Torpor typically emerged on day 3 [2-4], the probability of torpor increased with each day of calorie restriction, with 11.1% of trials resulting in torpor on day 1 (95% CI 3.7-24.1%) and 71.1% of trials resulting in torpor on day 5 (95% CI 55.7-83.6%) (Fig. 1*G–I*).

### Torpor is associated with increased neural activity in the POA and DMH

**Figure 2.**
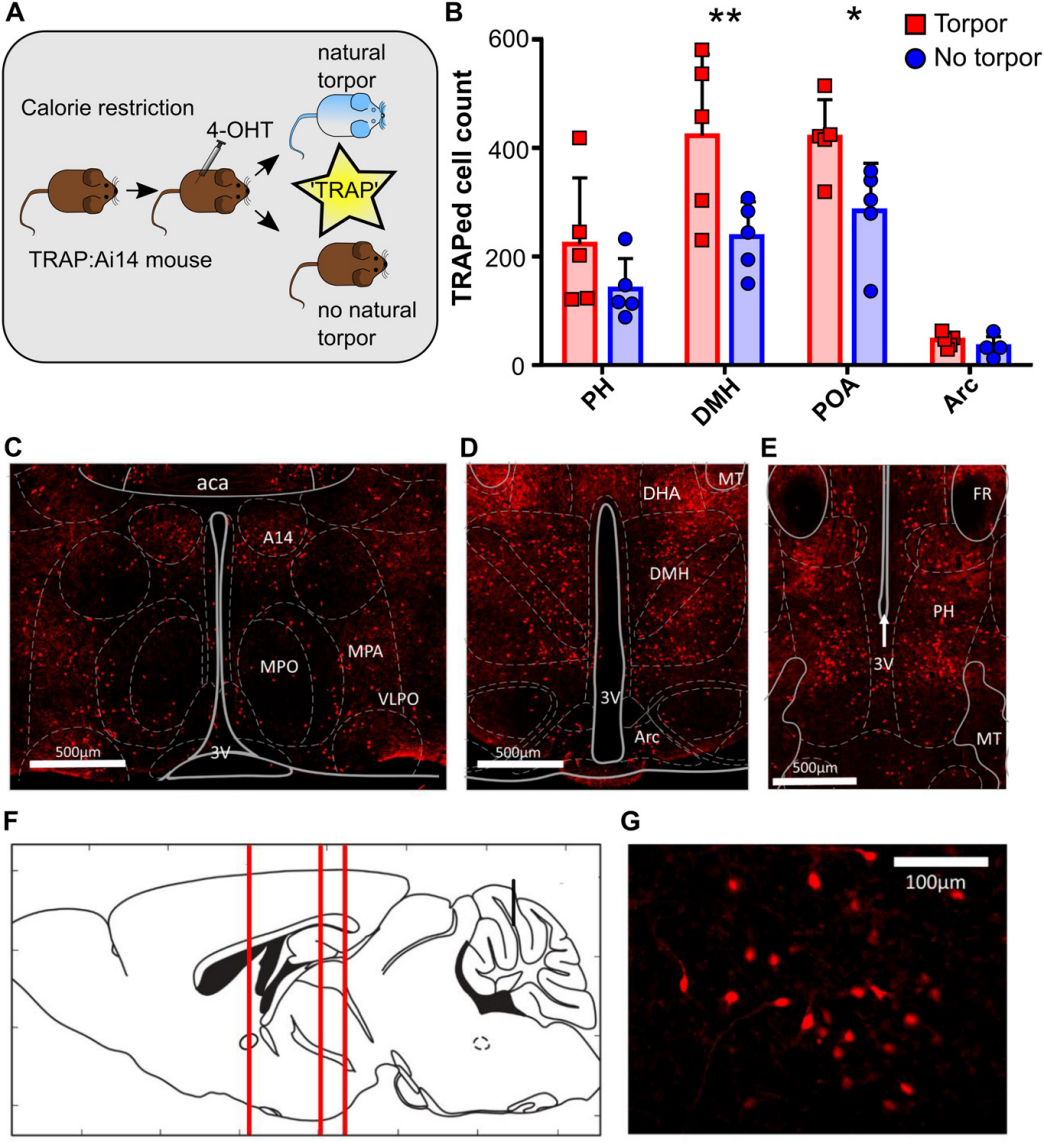
Torpor is associated with increased activity in dorsomedial and preoptic hypothalamus (*n* = 5 female *TRAP:Ai14* mice). ***A***, Torpor TRAP protocol for *TRAP2:Ai14* mice. Calorie-restricted *TRAP:Ai14* mice received 4-OHT at ZT7; those that entered torpor following the injection are compared with those that did not. ***B***, Counts of td-Tomato-positive cells by ROI in calorie-restricted mice that entered torpor following 4-OHT (red) or did not enter torpor following 4-OHT (blue). Torpor was associated with higher numbers of TRAPed cells in the DMH and POA compared with animals that did not enter torpor. Repeated-measures ANOVA comparing TRAPed cell count in mice that entered torpor with those that did not for each of the four ROIs. Main effect for torpor versus no torpor (*p* < 0.05). Main effect for ROI (*p* < 0.0001). Significant torpor versus no torpor by ROI interaction (*p* < 0.05). Holm–Sidak's multiple comparisons test: **p < 0.01; *p < 0.05. Data are mean and SD. ***C***, Coronal section showing POA neurons. ***D***, DMH and Arc neurons. ***E***, PH neurons. ***F***, Sagittal schematic showing corresponding anterior-posterior location of coronal sections ***C–E***. ***G***, High-magnification image showing DMH torpor-active neurons expressing td-Tomato. aca, Anterior commissure (anterior part); A14, A14 dopamine cells; Arc, arcuate nucleus; DHA, dorsal hypothalamic area; MPO, medial preoptic nucleus; MPA, medial preoptic area; PH, posterior hypothalamic nucleus; POA, preoptic area; VLPO, ventrolateral preoptic nucleus; 3V, third ventricle. *n* = 5 female mice per group.

To identify neuronal populations that were active during torpor, *TRAP:Ai14* mice were calorie-restricted and administered 4-OHT (50 mg/kg i.p.) on the third day at ZT7 to TRAP active neurons, with an ∼50% chance of each mouse entering torpor immediately after 4-OHT injection (*n* = 5 female mice). We observed widespread recombination resulting in tdTomato expression throughout the hypothalamus, both in mice that entered torpor (Torp^+^) following 4-OHT and those that did not (Torp^–^, Fig. 2).

Four hypothalamic areas were selected *a priori* for cell counting: POA, DMH, PH, and arcuate nucleus (arc). Entry into torpor was associated with increased tdTomato-labeled (“TRAPed”) cells in the DMH and POA compared with calorie-restricted mice that did not enter torpor (DMH Torp^+^ vs Torp^–^ mean difference 185.6 TRAPed neurons, CI 76-295, POA mean difference 135 TRAPed neurons, CI 26-245, Holm–Sidak multiple comparisons test, *p* < 0.01 and *p* < 0.05, respectively). In contrast, the arcuate and PH did not show significant differences between Torp^+^ and Torp^–^ mice (Fig. 2*B*). These differences in activity in the POA and DMH were not because of greater weight loss in the mice that entered torpor, indicating that the increased activity related to the occurrence of torpor per se rather than a response to the degree of calorie deficit (mean weight loss Torp^+^ 2.6 ± 0.7 g, vs 2.4 ± 0.3 g Torp^–^, *t*_(8)_ = 0.66, *p* = 0.46; percentage weight loss Torp^+^ 11.6 ± 2.3% vs 11.0 ± 1.2% Torp^–^, *t*_(8)_ = 0.57, *p* = 0.58).

### Chemoactivation of torpor-TRAPed DMH neurons

**Figure 3.**
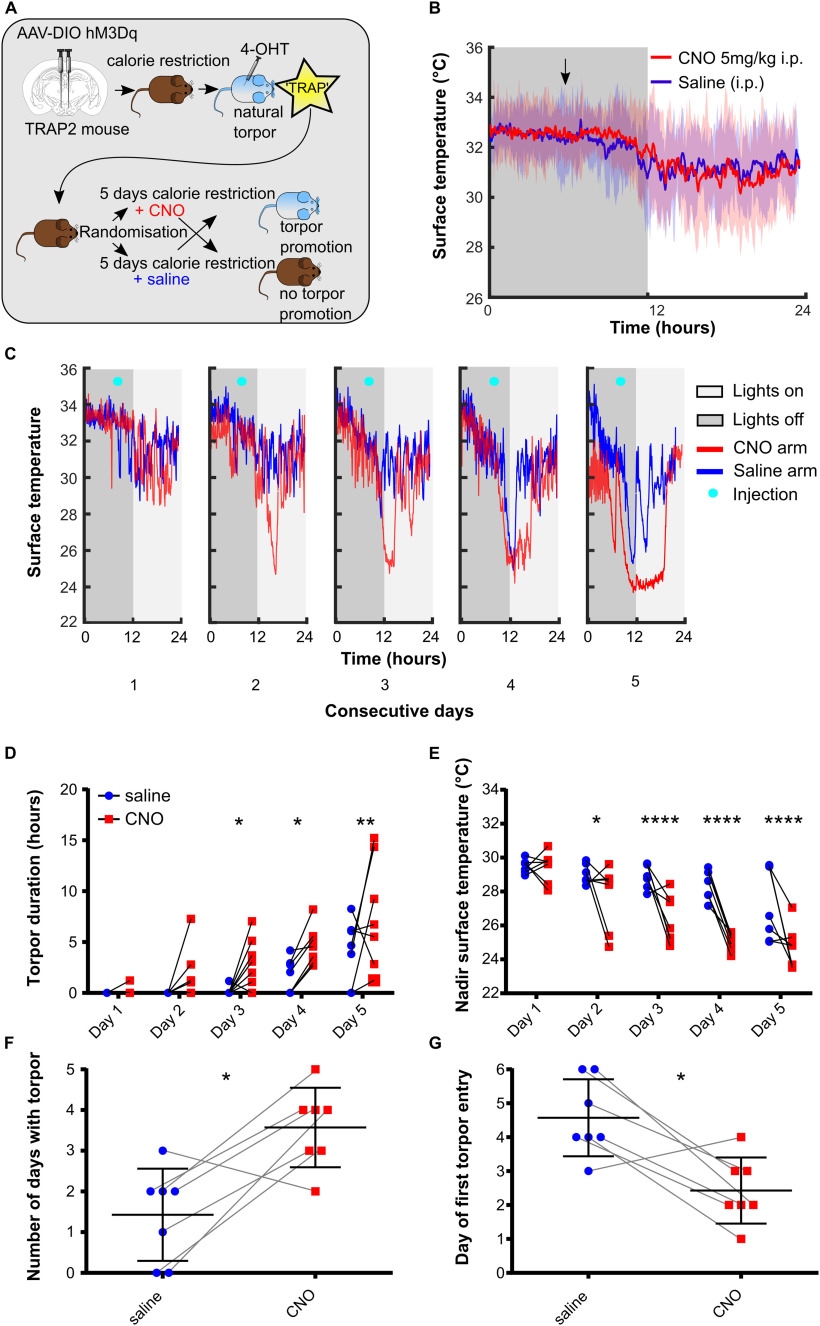
Chemoactivating DMH torpor-TRAPed neurons promote, prolong, and deepen torpor bouts in calorie-restricted mice (*n* = 7 female Torp^+^ hM3Dq mice). ***A***, DMH injection and torpor-TRAP protocol. ***B***, CNO does not trigger torpor in DMH torpor-TRAPed mice that are not calorie-restricted. Shaded area represents 95% CI. ***C***, Example plot showing surface temperature in calorie-restricted DMH torpor-TRAPed mice receiving CNO (red) or saline (blue) at 7 h after lights off (cyan marker). ***D***, CNO given to calorie-restricted DMH torpor-TRAPed mice increases the total time spent in torpor and decreases the nadir surface temperature reached (***D*** and ***E***, respectively). Two-way repeated-measures ANOVA, significant main effect for CNO versus saline trials: *p* < 0.05 for both torpor duration and nadir temperature. Holm–Sidak's multiple comparisons test: significant difference between CNO and saline on individual days (*p < 0.05, **p < 0.01, ***p < 0.001, and ****p < 0.0001, respectively). CNO increased the total number of days in which torpor occurred and resulted in torpor occurring after fewer days of calorie restriction (***F*** and ***G***, respectively). Wilcoxon matched-pairs signed rank test or paired *t* test, comparing number of torpor bouts and day of first torpor bout when mice received CNO versus saline (*p < 0.05).

**Figure 4.**
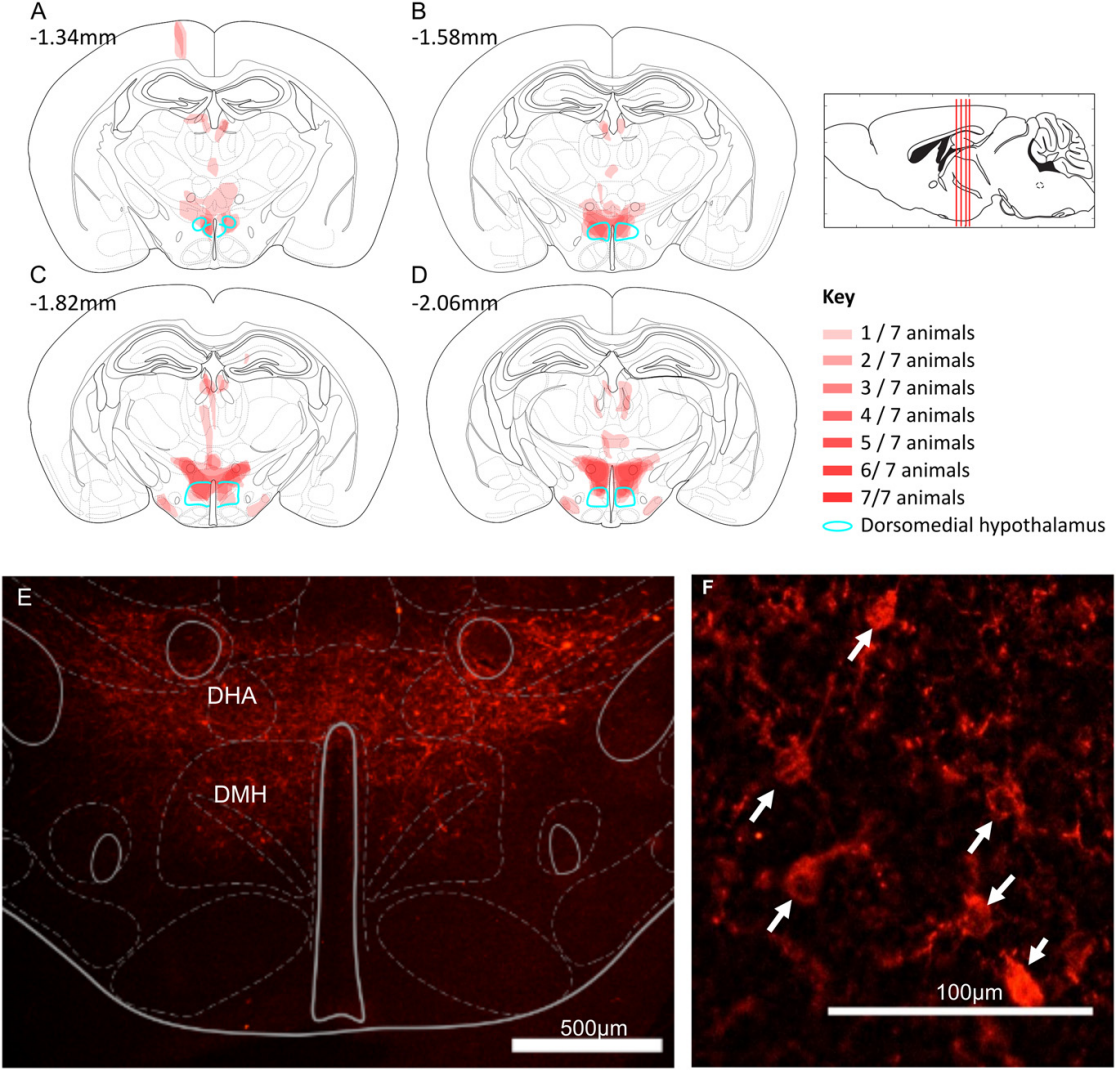
Torpor-TRAPed DMH neurons (*n* = 7 female Trap^+^ hM3Dq mice). ***A–D***, Mapped extent of mCherry-labeled cell bodies, indicating TRAPed cells expressing hM3DGq. TRAPed cells were observed in the DMH (marked in green) and dorsal hypothalamic area (DHA) of all mice. Injection tracts visible in ***A*** at bregma −1.34 mm, resulting in TRAPed cells in the cortex. Two mice also showed mCherry-labeled cell bodies in the medial tuberal nucleus (visible in ***C***,***D***). ***E***, ***F***, Example mCherry labeling (red) indicating DREADD expression in TRAPed cells in an hM3Dq-DMH-TRAP mouse. Expression in the DMH and DHA. Immunohistochemistry performed labeling the mCherry component of the hM3Dq-mCherry fusion protein. ***F***, Arrows indicate mCherry-labeled neuron cell bodies; 15 μm coronal sections.

To test whether neurons within the DMH play a causal role in torpor, we targeted expression of the excitatory DREADD hM3Dq to neurons in the DMH that were active during torpor. Nine *TRAP2* mice had injection of the Cre-dependent excitatory DREADD AAV (AAV2-hSyn-DIO-hM3Dq-mCherry) into the DMH (Fig. 4), followed by 5 d of calorie restriction with 4-OHT injection on d 5 (Fig. 3*A*). Of these mice, 7 entered torpor following 4-OHT administration and were included in subsequent experiments (Torp^+^ hM3Dq).

Chemoactivation of DMH torpor-TRAPed neurons by intraperitoneal injection of CNO to fed mice did not trigger torpor, nor did it alter surface temperature (Fig. 3*B*). However, CNO delivered each day at ZT7, to calorie-restricted mice, increased the probability of the mice entering torpor over 5 d (Fig. 3*C–G*). Torpor emerged after fewer days of calorie restriction when mice received CNO compared with when the same mice were calorie-restricted and received saline (first torpor bout appeared on day 2.4 ± 1.0 (CNO) vs 4.6 ± 1.1 (saline), paired *t*_(6)_ = 3.60, *p* < 0.05). There were more torpor bouts per mouse on the CNO arms compared with the saline arms (3.6 ± 1.0 (CNO) vs 1.4 ± 1.1 (saline) bouts, paired *t*_(6)_ = 3.60, *p* < 0.05). Likewise, when mice received CNO during the 5 d of calorie restriction, torpor bouts were longer than in the arms when they received saline (two-way repeated-measures ANOVA main effect for CNO vs saline, *F*_(1,6)_ = 8.14, *p* < 0.05). Similarly, during calorie restriction trials in which mice were given CNO, the daily nadir temperature reached was lower than when they were given saline (two-way repeated-measures ANOVA main effect for CNO vs saline, *F*_(1,6)_ = 12.2, *p* < 0.05).

The weights of mice on entry into the CNO versus saline arm of the trial were not different (24.7 ± 2.7 g (CNO) vs 24.7 ± 2.7 g (saline), paired *t*_(6)_ = 0.049, *p* = 0.96). Hence, the increased propensity to enter torpor observed with chemoactivation of hM3Dq-DMH-torpor-TRAP mice was not because of systematic differences in their weights.

### Chemoinhibition of torpor-TRAPed DMH neurons

Twelve *TRAP2* mice underwent injection of the Cre-dependent inhibitory DREADD (pAAV2-hSyn-DIO-hM4Di-mCherry) into the DMH followed by 5 d of calorie restriction with 4-OHT injection on d 5. Of these mice, 6 entered torpor following 4-OHT administration and were included (Torp^+^ hM4Di). These were evaluated for inhibition of torpor in response to CNO compared with saline.

**Figure 5.**
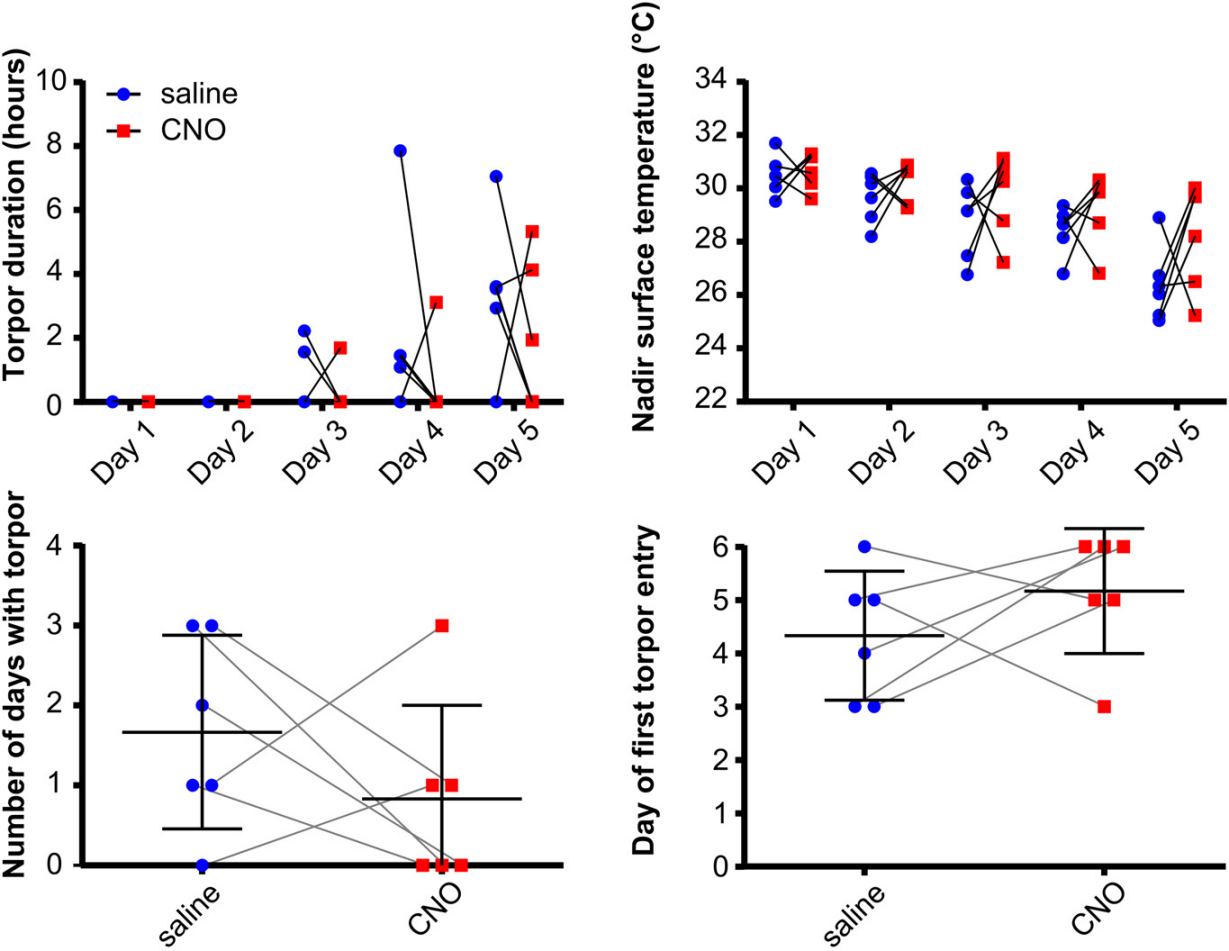
Chemoinhibition of DMH torpor-TRAPed DMH neurons does not affect torpor in calorie-restricted mice (*n* = 6 female Trap^+^ hM4Di mice). CNO did not affect the duration of torpor bouts or nadir surface temperature reached (two-way repeated-measures ANOVA, no main effect for CNO vs saline across all days, with Fisher's least significant difference test for CNO vs saline at each day of calorie restriction for each measure, *p* > 0.05 throughout). CNO did not affect the total number of days in which torpor occurred, nor the first day on which torpor occurred during 5 d of calorie restriction (paired *t* test, *p* > 0.05). *n* = 6 female mice.

Chemoinhibition of torpor-TRAPed DMH neurons in calorie-restricted mice did not reduce the total number of days in which torpor occurred (0.8 ± 1.2 (CNO) vs 1.7 ± 1.2 bouts (saline), paired *t*_(6)_ = 1.05, *p* = 0.34). Nor did it delay the onset of torpor across the 5 d of calorie restriction (torpor appeared on day 5.2 ± 1.2 (CNO) vs day 4.3 ± 1.2 d (saline), paired *t*_(5)_ = 1.05, *p* = 0.34) (Fig. 5). There were no differences between calorie restriction trials in which mice received CNO compared with when they received saline, in terms of time spent in torpor or nadir surface temperature reached (two-way repeated-measures ANOVA main effect for CNO vs saline, *F*_(1,5)_ = 1.37, *p* = 0.29 and *F*_(1,5)_ = 1.06, *p* = 0.35, respectively). Mouse weight on entry into the CNO arms was not different from that in the saline arms of the trial (25.5 ± 1.9 g (CNO) vs 25.2 ± 2.1 g (saline), paired *t*_(5)_ = 0.73, *p* = 0.5). Hence, chemoinhibition of Torp^+^ hM4Di mice did not alter the expression of torpor during 5 d of calorie restriction.

### Controls

**Figure 6.**
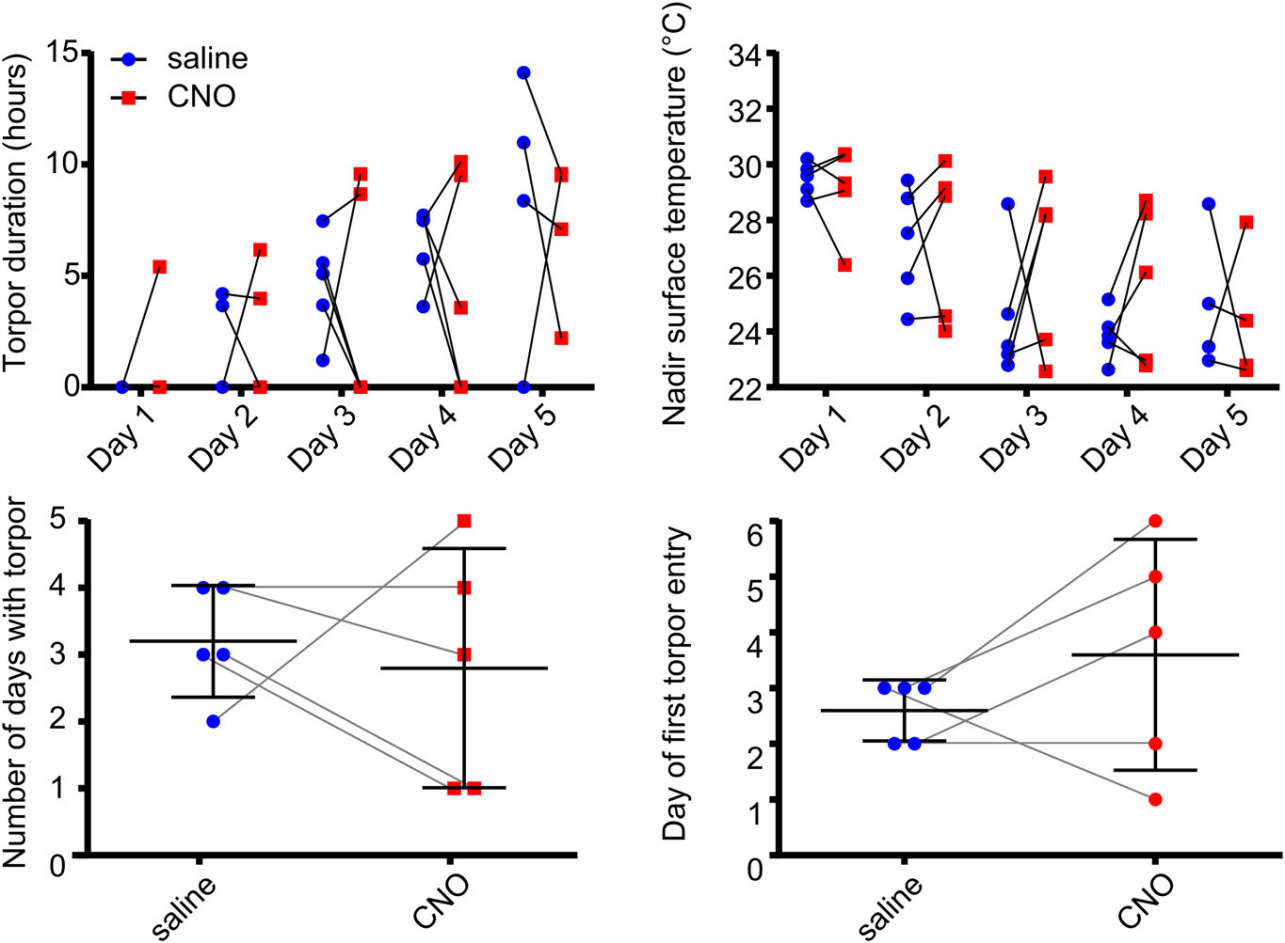
CNO administration does not affect torpor in DMH homecage-TRAPed mice. CNO did not affect the duration of torpor bouts or the nadir surface temperature reached (two-way repeated-measures ANOVA, no main effect for CNO vs saline across all days, with Holm–Sidak's multiple comparisons for CNO vs saline at each day of calorie restriction for each measure, *p* > 0.05 throughout). CNO did not affect the total number of days in which torpor occurred, nor the first day on which torpor occurred during 5 d of calorie restriction (paired *t* test, *p* > 0.05).

Five mice underwent hM3Dq vector injection into the DMH followed by 4-OHT at ZT7 with free access to food in the homecage (Homecage hM3Dq). They were then entered into the randomized, crossover design, calorie restriction trial receiving CNO, then saline, or vice versa. Chemoactivation of DMH neurons TRAPed in the fed homecage condition had no effect on torpor, in terms of the day of first torpor bout appearance (4 [1.5-5.5] CNO, vs 3 [2-3], Wilcoxon matched-pairs signed rank test, W = 6.0, *p* = 0.5, saline), total number of torpor bouts across 5 d calorie restriction (2.8 ± 1.8 vs 3.2 ± 0.84, paired *t*_(4)_ = 0.43, *p* = 0.69), daily nadir surface temperature (two-way repeated-measures ANOVA main effect for CNO vs saline, *F*_(1,4)_ = 0.20, *p* = 0.68), or daily torpor bout duration (two-way repeated-measures ANOVA main effect for CNO vs saline, *F*_(1,4)_ = 0.06, *p* = 0.83) (Fig. 6).

**Figure 7.**
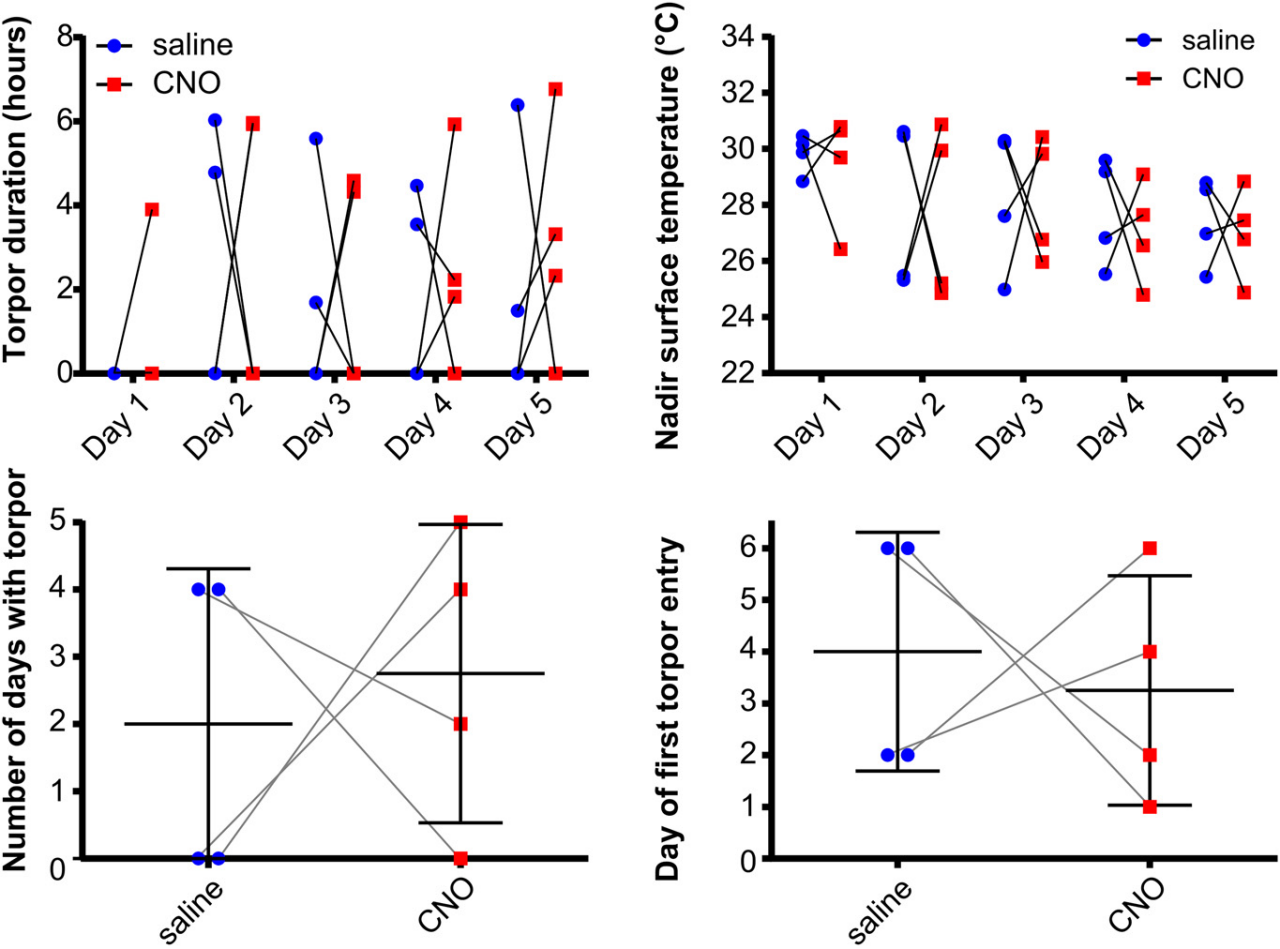
CNO administration does not affect torpor in WT mice (*n* = 4 female mice). CNO did not affect the duration of torpor bouts or the nadir surface temperature reached (two-way repeated-measures ANOVA, no main effect for CNO vs saline across all days, with Holm–Sidak's multiple comparisons test for CNO vs saline at each day of calorie restriction for each measure, *p* > 0.05 throughout). CNO did not affect the total number of days in which torpor occurred, nor the first day on which torpor occurred during 5 d of calorie restriction (paired *t* test, *p* > 0.05).

CNO injection had no effect on torpor in WT calorie-restricted control mice (*n* = 4), in terms of the day of first torpor bout appearance (3 [0.5-4.75] vs 2 [0-4], Wilcoxon matched-pairs signed rank test, W = −3.0, *p* = 0.75), total number of torpor days in which torpor occurred across the 5 d calorie restriction (3 [1.25-5.5] vs 4 [2-6], Wilcoxon matched-pairs signed rank test, W = 3.0, *p* = 0.75), daily nadir surface temperature (two-way repeated-measures ANOVA, *F*_(1,3)_ = 0.04, *p* = 0.85), or daily torpor bout length (two-way repeated-measures ANOVA, *F*_(1,3)_ = 0.10, *p* = 0.78) (Fig. 7).

### Phenotyping DMH torpor-TRAPed neurons

**Figure 8.**
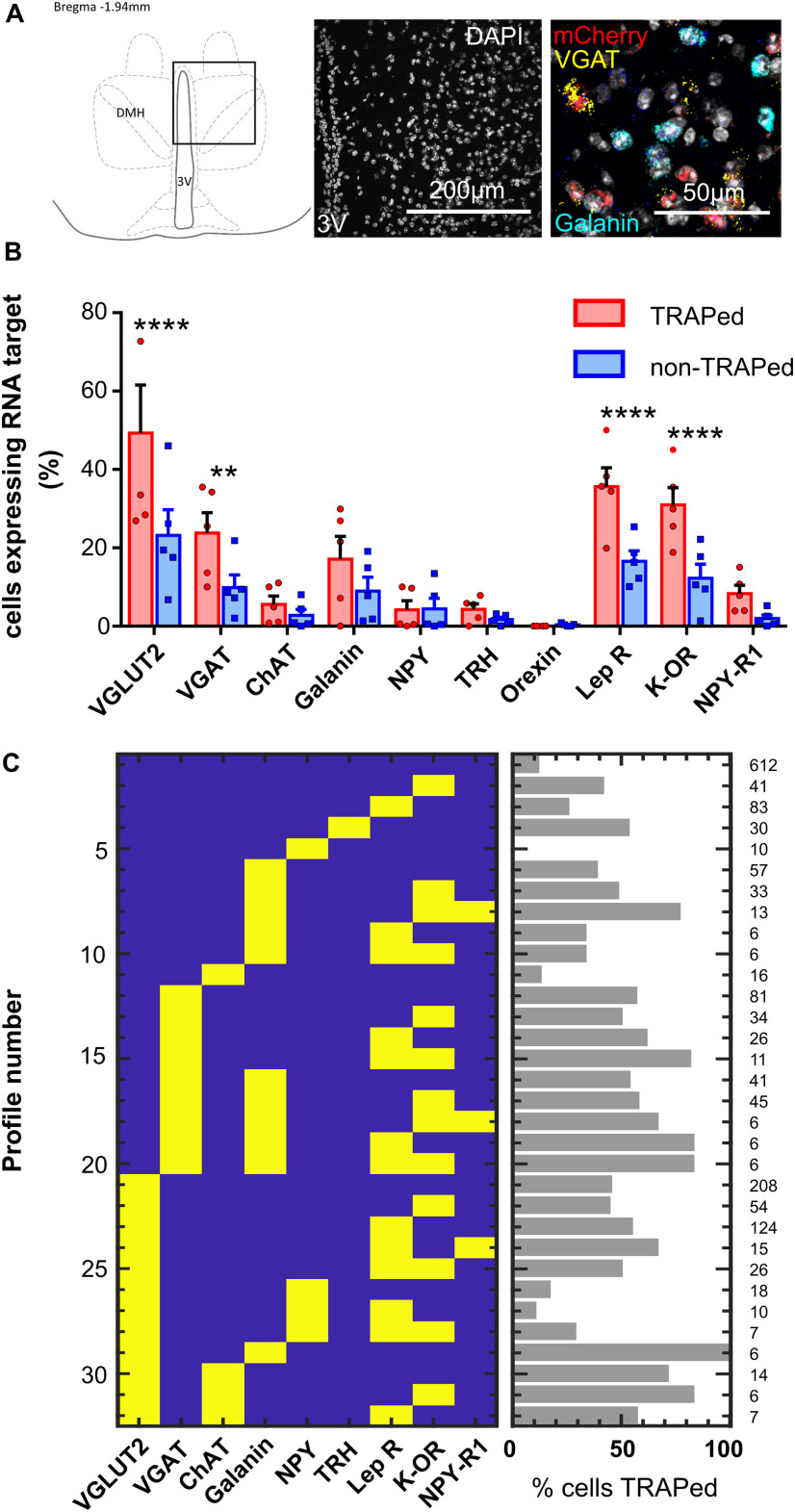
Multiplex RNA ISH (RNAscope) data (*n* = 5 Torp^+^ hM3Dq female mice). ***A***, Representative section showing DMH region analyzed with RNA ISH. ***B***, Proportion of TRAPed versus non-TRAPed cells that expressed each RNA target (two-way ANOVA with Holm–Sidak's multiple comparisons tests assessing difference in expression of the RNA target in TRAPed compared with non-TRAPed cells). **p < 0. 01. ****p < 0.001. ***C***, Patterns of RNA target expression observed in the DMH (excluding patterns that occurred in <5 cells) column on right indicates total number of cells expressing each profile.

Sections from Torp^+^ hM3Dq mice were selected for RNAscope analysis (*n* = 5 sections from 5 female mice, Fig. 8). One target probe, NeuN, did not produce a meaningful pattern of labeling. Because of the failure of NeuN, we were unable to exclude non-neuronal cells from the analysis.

A total of 1771 DAPI-stained nuclei were identified across five sections from 5 animals (345 ± 65 cells per animal). Four RNA targets were significantly more likely to be expressed in TRAPed cells compared with non-TRAPed cells: vesicular glutamate transporter 2 (VGLUT2), vesicular GABA transporter (VGAT), leptin receptor B, and κ-opioid receptor (two-way ANOVA with Holm–Sidak's multiple comparisons tests, *p* < 0.001, *p* < 0.01, *p* < 0.001, and *p* < 0.001, respectively, see Fig. 8). Among cells expressing these RNA targets, we found that VGLUT2 and leptin receptor were commonly coexpressed (38% of VGLUT2 cells also expressed leptin receptor compared with 21% that also expressed κ-opioid R), while VGAT and κ-opioid receptor were commonly coexpressed (38% of VGAT-expressing cells also expressed κ-opioid R, whereas 23% of VGAT cells expressed leptin receptor).

## Discussion

The data presented here support the hypothesis that both the DMH and the POA contain neurons that are active in the period leading up to a torpor bout. We show here that chemoactivation of DMH torpor-TRAPed neurons increases the probability, as well as the depth and the duration of torpor in calorie-restricted mice.

The POA has been implicated in the induction of torpor, or torpor-like states in mice (Hrvatin et al., 2020; Takahashi et al., 2020; Zhang et al., 2020). Previous studies have also identified that the DMH contains neurons that express c-fos around the time of torpor (Hitrec et al., 2019; Hrvatin et al., 2020), and several studies have demonstrated that projections from the POA to the DMH can decrease body temperature (Song et al., 2016; Zhao et al., 2017; Takahashi et al., 2020). However, a specific role for the DMH in the control of torpor, rather than a homeostatic thermoregulatory, or counter-regulatory role, has not been established. Here, by chemogenetically reactivating specifically those DMH neurons that expressed c-fos around the time of torpor, we have shown that they do indeed contribute to the generation of torpor.

Chemoactivation of DMH torpor-TRAPed neurons did not alter body temperature after single doses of CNO delivered to mice with free access to food. This suggests that the TRAPed neurons play a specific role in promoting torpor under calorie-restricted conditions, but that they are not sufficient to trigger it in the fed state. They likely form part of a chorus of signals that indicate negative energy balance and the need to engage torpor. If, on the other hand, the TRAPed neurons were simply part of a thermoregulatory circuit that inhibits thermogenesis under “normal” homeostatic conditions, then one might expect chemoactivation to produce a physiological response that is independent of calorie restriction.

Whether there is a single group of neurons capable of inducing torpor (i.e., a torpor “master switch”) remains unknown, although emerging evidence places the POA as a potential candidate (Hrvatin et al., 2020; Takahashi et al., 2020; Zhang et al., 2020). The observation of torpor-promoting effects following chemoactivation of DMH neurons supports the hypothesis that a network of regions contributes to torpor induction. The DMH would then be considered one part of this network. If neurons in the POA are sufficient to trigger torpor, then the DMH might form part of the afferent signal to the POA, or it might modulate the descending efferent signals from the POA.

Another question is whether torpor is triggered, maintained, and terminated by the activity of a single population of neurons, or whether different populations are each responsible for timing the different phases. The observation that chemoactivating DMH torpor-TRAPed neurons increases bout duration as well as increasing the likelihood of torpor occurring hints that torpor may be induced and maintained by the same population of neurons. If, on the other hand, the effect of chemoactivation of DMH torpor-TRAPed neurons was to increase the probability but not the duration of torpor (or vice versa), then this would support the hypothesis that these phases of torpor are governed by distinct neuronal populations.

The finding that activation of neurons with in the DMH contributes to torpor induction, a physiological response characterized by suppression of thermogenesis, is particularly interesting. Within the framework of our current understanding of hypothalamic thermoregulatory circuits, the DMH generally emerges as a driver rather than inhibitor of thermogenesis, at least in rodents (Rezai-Zadeh et al., 2014; Zhao et al., 2017; Morrison and Nakamura, 2019), although cholinergic neurons in the DMH may suppress brown adipose tissue thermogenesis (Jeong et al., 2015). In contrast, the DMH neurons TRAPed during torpor in this study appear to contribute to the suppression of thermogenesis associated with torpor, supporting a more complex and bidirectional role for the DMH in temperature regulation (Saper and Machado, 2020).

We used RNAscope to explore the phenotype of torpor-TRAPed cells within the DMH. We appear to have predominantly TRAPed two populations of neurons: one expressing VGLUT2 and leptin receptor B, and the other expressing VGAT and κ-opioid R. The VGAT and κ-opioid receptor-expressing cells might represent a population of neurons that project to the raphe pallidus to inhibit thermogenesis during torpor (Hitrec et al., 2019). Indeed, central blockade of κ-opioid receptors blocks the hypothermic response to calorie restriction in male mice (Cintron-Colon et al., 2019). The VGLUT2 and leptin receptor-expressing neurons are also interesting. A similar population have been described previously, as having a role in thermogenesis and maintenance of stable body weight (Zhang et al., 2011; Rezai-Zadeh et al., 2014). If the glutamatergic/leptin receptor-expressing population we have TRAPed is the same as those previously identified, then these neurons might have a role in thermogenesis during the emergence from torpor. Alternatively, they might represent a novel population of leptin-sensitive neurons with a role in torpor promotion.

Finally, we note that a population of neurons that express genes not targeted in our selection of RNA probes might have been critical to the promotion of torpor presented here. For example, we did not include other VGLUT subtypes, and we found 7% of cells that expressed mCherry expressed no other RNA target. These might be important contributors to the promotion of torpor observed in our experiments. The failure of the RNA NeuN probe in our hands limits interpretation of the cell phenotyping data because non-neuronal cells could not be excluded from the analysis and so these findings should be regarded as preliminary and hypothesis-generating for future studies.

### Limitations

We opted to deliver 4-OHT in anticipation of torpor, in an effort to reduce the likelihood of arousing the mice and TRAPing arousal circuits. It is possible that this approach resulted in TRAPing neurons within the DMH that are not involved in torpor induction, and/or missed populations whose activity increased nearer to the point of torpor induction. Hence, it remains possible that delivery of 4-OHT during torpor might TRAP a population of DMH neurons capable of inducing torpor in the absence of calorie restriction.

We did not find that chemoinhibition of DMH torpor-TRAPed neurons prevented torpor induction in calorie-restricted mice. It is worth noting that incomplete suppression of neuronal firing with the inhibitory DREADD, hM4Di, has been reported (Smith et al., 2016). Therefore, this result could reflect failure to inhibit these DMH neurons sufficiently to prevent their contribution to torpor. Alternatively, it might be necessary to silence a wider population of neurons, including potentially those in other brain regions, to prevent torpor.

Finally, we used female mice in our experiments because in our experience they have a greater tendency to enter torpor. Whether our results are generalizable to male mice and whether the stage of the ovarian cycle interacts with the expression of torpor are currently unknown. Recent data suggest that neurons in the POA that express estrogen receptors may have a role in torpor expression, although the effect on torpor of modulating the activity of these estrogen receptors has not been established (Zhang et al., 2020, 2021). We do not believe that the delivery of 4-OHT during the TRAPing process influenced the results observed here (and our findings are in line with those of Hrvatin et al., 2020, who also used 4-OHT). 4-OHT is generally considered an estrogen receptor antagonist (Shiau et al., 1998), and we observed no effects on torpor propensity in control mice that received 4-OHT under fed homecage conditions.

In conclusion, our data indicate that neurons within the DMH promote torpor entry and increase the duration and depth of torpor bouts in calorie-restricted mice. Chemoactivating these neurons in fed mice neither triggered torpor nor altered thermoregulation. Hence, we hypothesize that the DMH plays a modulatory role capable of promoting torpor. Important future work should clarify the phenotype of these neurons and investigate their interaction with other areas believed to be involved in torpor, such as the POA.
